# Remote psychotropic medication advice for general practitioners: a quality improvement project

**DOI:** 10.1192/bjo.2021.568

**Published:** 2021-06-18

**Authors:** Emily Rackley, Rosemary King

**Affiliations:** Gloucestershire Health and Care NHS Foundation Trust

## Abstract

**Aims:**

Our first aim was to first find out how confident general practitioners were about referring in to the Gloucester Recovery Team and managing psychotropic medications. Our second aim was to then improve general practitioner's self-rated scores of confidence in managing psychotropic medication whilst also improving general practitioner's satisfaction with waiting times for patient's referred to the Gloucester Recovery Team.

**Method:**

We planned to introduce an email address for GPs to use to seek medication and diagnostic advice for patients known to and not known to the Recovery Team. We initially introduced this for the ‘Team 2’ catchment area consisting of five practices within Gloucester. These were then read and replied to by the Team 2 consultant, Dr Ikram, as appropriate. A further survey was then sent out.. These results provided both quantitative ordinal data through a likert scale, which was then transformed into binomial data, such as those scoring ‘extremely confident’ ‘very confident’ ‘somewhat confident’ vs ‘not so confident’ and ‘not confident at all’ which is then compared using relative risk.

**Result:**

Our response rate for our initial survey was 8 general practitioners, and for our follow-up survey 1 general practitioner and 2 nurse prescribers. Confidence in continuing psychotropic medications increased from 7 out of the 8 (78%) stating somewhat confident to extremely confident to 3 out of the 3 (100%) after the introduction of the email; a relative change of 1.14 (95% confidence interval 0.87-1.48 p = 0.318). Confidence in initiating psychotropic medications increased from 4 out of the 8 (50%) stating somewhat confident to extremely confident to 2 out of the 3 (66%) after the introduction of the email; a relative change of 1.33 (95% confidence interval 0.46-3.84 p = 0.594).

**Conclusion:**

Analysing the qualitative data showed the email address was used for a variety of requests and advice including: 1) A capacity assessment, 2) Initiating medications for depression and anxiety, 3) Medications during pregnancy, 4) Medication for those with Intellectual Disability, 5) Switching medication, 6) Medications for poor sleep and 7) Mood stabilising medication.

This change appeared to be well received, however the response rate was very low which makes full analysis difficult. We also included nurse practitioners working in primary mental health in our second survey, whereas the initial survey was only sent to GPs. This initiative was also only started for 5 of the GP practices within Gloucester, and there may be a different knowledge base/confidence amongst the other practices.

